# A retrospective survey of quality of reporting on randomized controlled trials of metformin for polycystic ovary syndrome

**DOI:** 10.1186/1745-6215-15-128

**Published:** 2014-04-17

**Authors:** Baoying Chen, Jian Liu, Chun Zhang, Minyan Li

**Affiliations:** 1 Department of Traditional Chinese Medicine, The First People’s Hospital of Shunde, Foshan, PR China

**Keywords:** Metformin, Quality of reporting, Randomized controlled trials, Polycystic ovary syndrome, The CONSORT Statement

## Abstract

**Background:**

From previous reviews, there still have been controversies over the effect of metformin (MET) on reproductive function in PCOS patients. The reasons for the inconsistent findings especially lie in the transparency and accuracy of randomized controlled trials (RCTs) reports. However, we could find no data about the quality of RCTs reporting in MET for PCOS. Thus, a retrospective survey related to the quality of reporting in MET for PCOS was conducted.

**Methods:**

A retrospective survey was conducted by two investigators. Two investigators assessed the quality of overall reporting and key methodological factors reporting using items from the CONSORT 2010 statement.

**Results:**

A total of 39 RCTs were included in full text. The median overall quality score was 9, with a minimum of 2 and a maximum of 13. Good or general reporting existed in 11 items with positive rate of more than or equal to 50%. The median score of key methodological items was 4 with a minimum of 0 and a maximum of 5. Randomization, allocation concealment, blinding, baseline characteristics and intention-to-treat (ITT) analysis were reported in 26 (67%), 19 (49%), 20 (51%), 38 (97%) and 17 (44%) of the 39 RCTs, respectively. After adjustment, the mean overall score increased by about 1.71 for manuscripts with funding source (95% CI, 0.18 to 3.24), while it increased by about 3.51 for manuscripts published in one year increment (95% CI, 1.82 to 5.19). There was a relatively close, significant correlation (*r* = 0.589, *P* < 0.001) between the score of overall reporting quality and year of publication.

**Conclusion:**

Although the overall reporting quality of RCTs in MET for PCOS has improved over time, reporting of key methodological items remains poor. Reporting of RCTs on MET for PCOS should keep up with the standards of the CONSORT statement.

## Background

Polycystic ovary syndrome (PCOS) is characterized by chronic anovulation (failure or absence of ovulation) and hyperandrogenism (excessive production of male hormones in women) with clinical manifestations of irregular menstrual cycles, infertility, hirsutism, and acne [1], which is a common condition affecting women of reproductive age in 5 to 10% [2].

Administration of clomiphene citrate (CC) is the standard treatment for PCOS patients with anovulatory infertility. However, clomiphene resistance (failure to ovulate after taking clomiphene) is common, occurring in approximately 15 to 40% of women with PCOS [3]. In 2008, it was reported that insulin resistance was a significant contributor to the pathogenesis of PCOS [4]. Because of the insulin resistance in the pathogenesis of PCOS, metformin (MET), a biguanide and insulin-sensitizing drug used in the treatment of type 2 diabetes mellitus, was applied to the treatment of infertile women with PCOS before or during the ovulation induction [5].

Several systematic reviews and meta-analyses [6-13] have evaluated the efficacy of metformin in the treatment of anovulation because of PCOS. Most of these reviews concluded that metformin monotherapy represented a safe and valid therapeutic option for improving ovulation in PCOS patients. One review [13] concluded that combination of MET and CC could gain advantage over a single administration in the ovulation induction and pregnancy rate, but another review [12] concluded this combination was no better than monotherapy (MET alone or CC alone). Moreover, there still have been controversies over the effect of MET on reproductive function in PCOS patients [14-17].

Randomized controlled trials (RCTs) are widely accepted as the ‘gold standard’ for accumulating strong evidence for any health care intervention. Moreover, quality of reporting is essential for guiding journal peer-review decisions and experts’ recommendations, conducting unbiased meta-analysis and influencing our interpretation of evidence [18]. The reasons for the inconsistent findings of above reviews lie in the bias of literature search and screening and especially the transparency and accuracy of RCT reports. The Consolidated Standards of Reporting Trials (CONSORT) statement is an international consensus expert guideline developed in 1996 and last updated in 2010, aimed at improving the reporting quality of published RCTs [19]. The CONSORT is widely accepted in the field of clinical trials and is supported by a growing number of health care journals and editorial groups.

However, we could find no data about the quality of RCTs reporting in MET for PCOS. Thus, a retrospective survey related to the quality of reporting in MET for PCOS was conducted. The aim of this study was to assess the overall quality of published articles of randomized trials in MET for PCOS with a special focus on the key methodological items that safeguard against biases, namely appropriate randomization, allocation concealment, blinding, baseline characteristics and analysis according to intention-to-treat (ITT) principle. Secondarily, we also aimed at determining factors associated with better reporting quality.

## Methods

### Search strategy

A systematic and comprehensive literature search was conducted with the aim of identifying published prospective RCTs of MET for PCOS. No resources were available to search literature published in languages other than English.

The following databases were searched from their inception through February 2013: MEDLINE, EMBASE, the Cochrane Central Register of Controlled Trials (CENTRAL), and CINAHL.

Keywords were approved by all the authors and included ‘PCOS’, ‘polycystic ovary syndrome’, ‘metformin’, ‘MET’, ‘clomiphene citrate’, ‘CC’, ‘randomized trials’, ‘RCT’. Eligible articles were identified by successive screening of titles and abstracts. Then the references section of each printed article was screened to identify any additional eligible articles.

### Inclusion and exclusion criteria

Types of studies: only RCTs of MET for PCOS were identified and selected for the analysis. Specifically, retrospective, non-randomized, cross-over RCTs, case-control, and quasi-randomized trials, abstracts in conference and case reports/series were excluded.

The criteria in terms of PCOS diagnosis had to be consistent with those as follows: oligo- or anovulation, clinical or biochemical signs of hyperandrogenism and polycystic ovaries visible with ultrasound. The RCTs on the effects on MET in patients who received gonadotrophins for IVF and not-IVF cycles were also included. Several different types of interventions were analyzed: MET versus placebo, MET versus CC, MET plus CC versus CC, MET plus CC versus MET. We also included trials in which MET combined with other interventions as the treatment group (for example, MET plus lifestyle versus placebo plus lifestyle, MET plus rFSH versus rFSH). The primary comparison was always between MET and the other treatment. Outcomes included live birth rate, rates of ovulation, pregnancy, abortion and discontinuation for adverse events.

### Assessment of reporting quality

#### Rating of overall reporting quality

An overall quality score with 13 items from the CONSORT 2010 statement was used (Table 1). Each item was scored 1 if it was reported and 0 if it was not clearly, or definitely not stated. These were among the items selected by previous evaluation studies of the CONSORT statement [20-23].

**Table 1:** Overall quality of reporting rating using items from the CONSORT statement (n = 39)

**Item**	**Criteria**	**Description**	**Number. of positive trials**	**%**	**95% CI**	**Cohen’s *к *** **coefficient**	**95% CI**
1	‘Randomized’ in the title or abstract	Study identified as a randomized controlled in the title or abstract	36	92	84 to 100	1	1
2	Background	Adequate description of the scientific background and explanation of rationale	38	97	92 to 100	0.62	0.35 to 0.95
3	Trial design	Description of trial design (such as parallel, factorial) including allocation ratio	34	87	76 to 98	0.74	0.52 to 0.98
4	Participants	Description of the eligibility criteria for participants	36	92	84 to 100	0.92	0.83 to 1.00
5	Interventions	Details of the interventions intended for each group	35	90	80 to 100	0.63	0.42 to 0.98
6	Outcomes	Definition of primary (and secondary when appropriate) outcome measures	23	59	43 to 75	0.81	0.65 to 0.99
7	Sample size	Description of sample size calculation	19	49	32 to 65	0.78	0.54 to 0.97
12	Statistical methods	Description of the statistical methods used to compare groups for primary outcomes, subgroup analyses, or adjusted analyses	29	74	60 to 89	0.68	0.43 to 0.96
13	Flow chart	Details on the flow of participants through each stage of the trials (number of patients randomly assigned, receiving intended treatment, completing the protocol and analyzed)	21	54	37 to 70	0.93	0.85 to 1.00
14	Recruitment	Dates defining the periods of recruitment and follow-up	20	51	35 to 68	0.54	0.36 to 0.92
17	Outcomes and estimation	For each primary and secondary outcome, a summary of results for each group is given, and the estimated effect size and its precision (for example, 95% CI)	20	51	35 to 68	0.86	0.73 to 0.99
18	Ancillary analyses	Clear statement of whether subgroup/adjusted analyses were prespecified or exploratory	20	51	35 to 68	0.66	0.31 to 0.97
19	Harms	Description of all important adverse events in each group	16	41	25 to 57	0.73	0.62 to 0.96

#### Rating of key methodological items

Five key methodological categories of randomization, allocation concealment, blinding, baseline characteristics and ITT analysis have been assessed separately because they relate to potential sources of bias [24-26]. We then developed eight ‘yes’/‘no’ items (Table 2), wording so that emphasis was placed on quality of reporting rather than adequacy of trial design. Each item was scored 1 if the method was appropriate and 0 if inappropriate or if the reporting was unclear.

**Table 2:** Reporting quality of key methodologic items (n = 39)

**Item**	**Criteria**	**Description**	**Number of positive trials**	**%**	**95% CI**	**Cohen’s *к *** **coefficient**	**95% CI**
8	Randomization	Description of the method used to generate the random sequence	26	67	51 to 82	0.83	0.72 to 0.98
9 and 10	Allocation concealment and implementation	Description of the method used to implement the random allocation sequence assuring the concealment until interventions are assigned	19	49	31 to 64	0.71	0.55 to 0.94
11	Blinding	Whether or not participants, those administering the interventions, or those assessing the outcomes were blinded to group assignment	20	51	27 to 55	0.72	0.48 to 0.95
15	Baseline data	An outline of baseline demographic and clinical characteristics of each group	38	97	92 to 100	0.65	0.49 to 0.97
16	Intention-to-treat analysis	Number of participants in each group included in each analysis and whether it was done by ‘intention-to-treat’	17	44	27 to 60	0.92	0.80 to 0.99

#### Data extraction

One of the investigators (Minyan Li) looked at the title, abstract and methodology of all the published papers to identify them as RCTs. General information (year of publication, journal of publication, impact factor of journal, collaboration research of different countries, region in which trials were conducted, funding source, choice of comparator interventions) were extracted (by Chun Zhang). Relevant studies were then photocopied with the author’s names, date and institution excluded for following rating by two assessors (Baoying Chen and Jian Liu). Two independent assessors (Baoying Chen and Jian Liu) blinded to each other’s ratings, completed the rating form independently. Cohen’s *к*-statistic was calculated to assess agreement between two assessors. Agreement was judged as poor if *к ≤* 0.20; fair if 0.20 lower than *к ≤* 0.40; moderate if 0.40 lower than *к ≤* 0.60; substantial if 0.60 lower than *к ≤* 0.80; good if *к* higher than 0.80; and perfect if *к =* 1 [20]. Discrepancies were reviewed in detail and subsequently settled by consensus.

### Data analysis

The characteristics of the publications, scores of overall reporting quality and five methodological items were then described by descriptive analysis. To identify factors associated with the overall quality of publications, we used this overall score as the outcome variable and the characteristics of the publications as independent variables which was modeled using linear regression. Only variables that were significant at *P ≤* 0.10 in the univariate models were used in a multivariable regression model for selecting significant variables. Variables significant at the 5% level in the final multivariable model were considered as significant predictors. To analyze the relationship between the score of overall reporting quality and year of publications, scatter plot and Pearson correlation analysis were performed.

To identify factors associated with methodological quality, we used this methodological score as the outcome variable in the regression analyses. As the outcome variable can be considered as a count, we relied on a Poisson regression model and adjusted the variance empirically. Descriptive statistical analysis, linear regression analysis, scatter plot and Pearson correlation were performed using SPSS version 20.0 (SPSS, Chicago, IL, USA). Analysis of Poisson regression model and Cohen’s *к*-statistics were performed using the SAS software, version 9.1 (SAS Institute, Inc, Cary, NC, USA). Database of RCTs in MET for PCOS are provided in Additional file 1.

## Results

**Figure 1:**
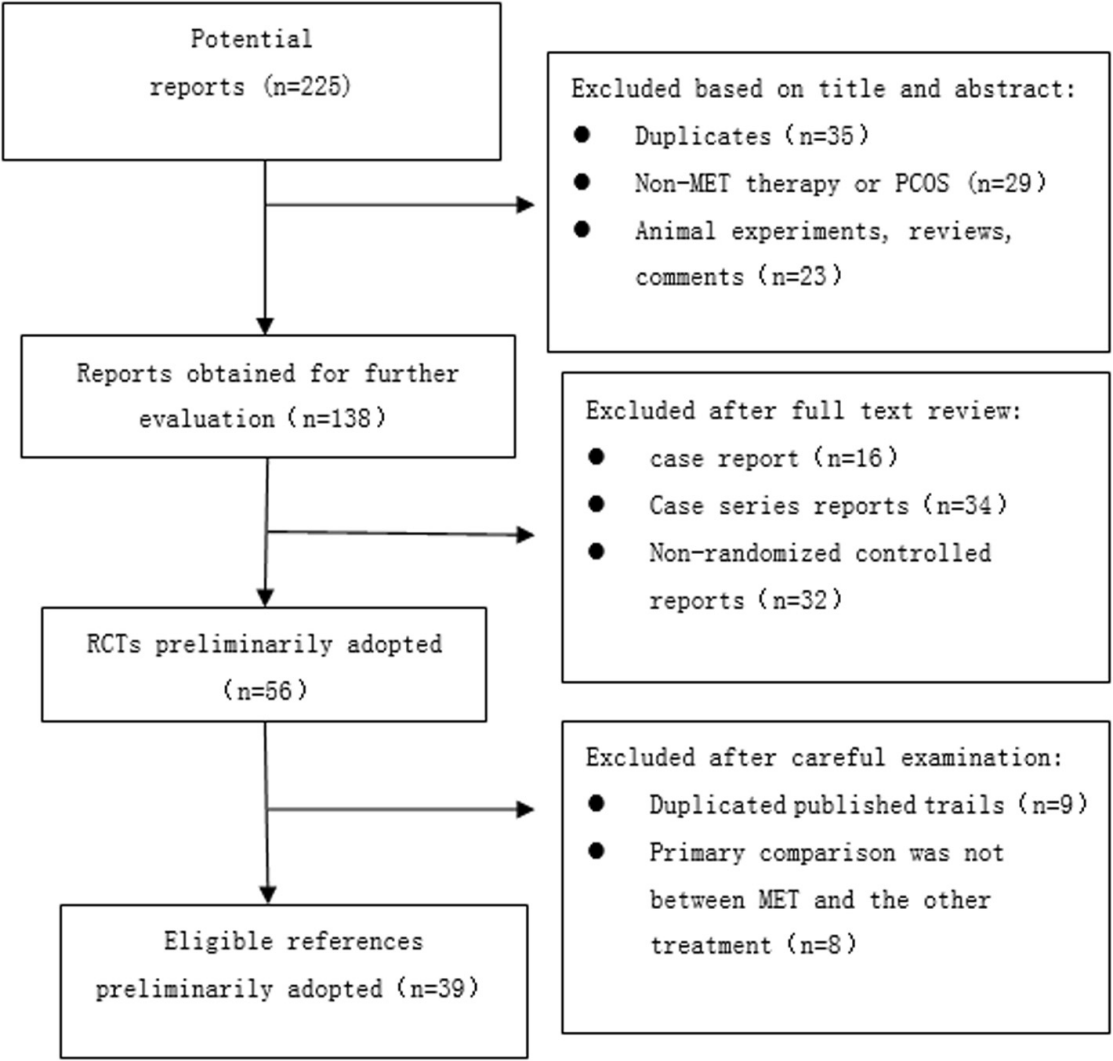
Flowchart of the article selection process.

The RCTs selection process is outlined in Figure 1. The researchers applied the search method to find 225 reports related to the topic, among which 35 reports of duplicates, 29 reports of non-MET therapy or PCOS, 23 reports of animal experiments, reviews and comments are excluded. One hundred and thirty-eight reports were obtained for further evaluation. Then researchers viewed the full text of all potentially eligible reports were obtained and picked out 16 case reports, 34 case series reports and 32 non-randomized controlled reports. Then, 56 RCTs preliminarily were adopted. After carefully reselecting, we pick out nine duplicated published reports and eight reports not comparing MET and the other treatment. A total of 39 relevant RCTs were included in the final analysis.

### Characteristics of included trials

The characteristics of RCTs included in the final analysis are described in Table 3. Counting the number of articles, frequency, which refers to RCTs of MET for PCOS, was found to be increasing over time: from 7 (17.9%) in 1996 to 2001 to 20 (51.3%) in 2008 to February 2013. The percentages of RCTs from North America and Europe were 15.4% and 41.0%. More than half of included trials neither got funding nor collaborated with researchers from different countries. Sixteen RCTs (41.0%) chose placebo as the comparator intervention. The RCTs were published predominantly in three journals, namely *The Journal of Clinical Endocrinology & Metabolism* (23.1%)*, Fertility and Sterility* (17.9%) and *Human Reproduction* (15.4%).

**Table 3:** Characteristics of included randomized controlled trials (RCTs) in metformin (MET) for polycystic ovary syndrome (PCOS)

**Features of included RCTs**	**Number of studies (n = 39)**	**%**
Year of publication		
1996 to 2001	7	17.9
2002 to 2007	12	30.8
2008 to February 2013	20	51.3
Revised CONSORT 2001		
Before	7	17.9
After	32	82.1
Regions in which RCTs were conducted		
North America	6	15.4
Europe	16	41.0
Others	17	43.6
Sources of trial funding		
Yes	19	48.7
No	20	51.3
Collaboration of different countries		
Yes	8	20.5
No	31	79.5
Choice of comparator interventions		
Placebo	16	41.0
Sole intervention	11	28.2
MET plus other therapies	3	7.7
Some of above combinations	9	23.1
Journals with most frequently published		
*The Journal of Clinical Endocrinology & Metabolism*	9	23.1
*Fertility and Sterility*	7	17.9
*Human Reproduction*	6	15.4
*The New England Journal of Medicine*	3	7.7
Eleven other journals^a^	14	35.9
Impact factors of included journal^b^		
0.00 to 2.99	10	25.6
3.00 to 5.99	24	61.5
6.00-	4	10.3

Abbreviations: *CONSORT*, Consolidated Standards of Reporting Trials; *MET*, metformin; *PCOS*, polycystic ovary syndrome; *RCT*, randomized controlled trial.

^a^Each journal published fewer than three RCTs.

^b^One journal (*Acta Med Indones*) did not have an impact factor.

### Quality of reporting

#### Rating of overall reporting quality

The ratings of overall quality of reporting are listed in Table 1. When the 39 RCTs were considered, the median overall quality score was 9, with a minimum of 2 and a maximum of 13. Good or general reporting existed in 11 items with positive rate of more than or equal to 50%, while items of ‘sample size’ and ‘harms’ presented less good reporting with positive rates of less than 50%.

Inter-rater agreements are reported for each item in Tables 1 and 2. A substantial, good, or perfect agreement was observed for 17 of 18 items. The inter-rater agreement was considered as moderate for item 14 (28 February 2013).

#### Rating of Key Methodological Items

Randomization, allocation concealment and implementation, blinding, baseline characteristics and ITT analysis were reported in 26 (67%), 19 (49%), 20 (51%), 38 (97%) and 17 (44%) of the 39 RCTs, respectively (Table 2). The median score of key methodological items was 4 with a minimum of 0 and a maximum of 5. Among the 39 studies, 1 (3%) did not report any of the five key methodological items (Table 2).

#### Exploratory analysis: factors associated with better reporting quality

In univariate analyses, year of publication and funding source were associated with an increased overall score. After adjustment, the multivariable linear regression model suggested that these two factors remained significant predictors of overall quality. Moreover, the mean overall score increased by about 1.71 for manuscripts with funding source (95% CI, 0.18 to 3.24; *P* < 0.05), while it increased by about 3.51 for manuscripts published in one year increment (95% CI, 1.82 to 5.19; *P* < 0.001) (Table 4).

**Table 4:** Multivariable linear regression analysis for factors associated with better overall score from the CONSORT statement (n = 39)

**Variables**	$\hat{\beta }$	**SE**	***t***	***s***	**95% CI**
Constant	5.52	0.81	6.80	< 0.001	3.87 to 7.17
Funding source	1.71	0.75	2.27	0.030	0.18 to 3.24
Year of publication	3.51	0.83	4.22	< 0.001	1.82 to 5.19

With regard to the methodological score, using univariate Poisson regression, no variable was included in the model.

### Trends of reporting quality

**Figure 2:**
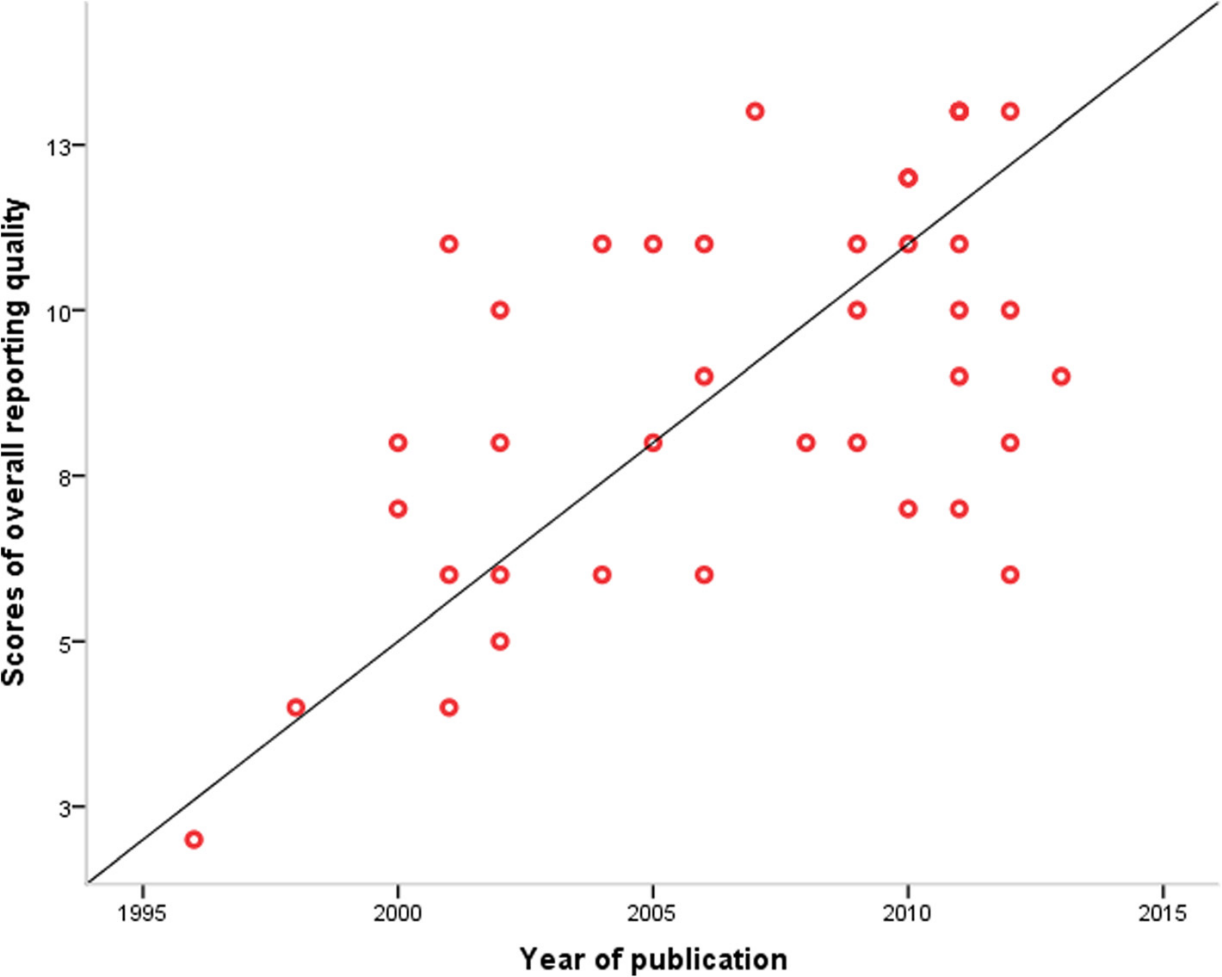
Correlation between the score of overall reporting quality and year of publications.

From Table 4, we saw the factor of year of publication is a powerful predictor for overall reporting quality with its coefficient 3.51. We want to know how the trend of overall reporting quality in RCTs of MET for PCOS will be with year increasing. There was a relatively close, significant linear correlation (r = 0.589, *P* < 0.001) between the score of overall reporting quality and year of publications (Figure 2).

## Discussion

This study demonstrated that the quality of reporting in RCTs on MET for PCOS was suboptimal especially in key methodological items. This indicated that RCTs on MET for PCOS syndrome needed improvement to meet the level of ‘reporting quality’ required by the CONSORT statement. It is important to follow the guidelines of the CONSORT statement for RCTs on MET for PCOS for two reasons. First, inconsistent findings of reviews [12,13] or controversies [14-17] still existed in the effect of MET on reproductive function in PCOS patients. Transparency and accuracy of RCT reports will benefit the evidence-based information extracting, assessing the validity of the results and medical decision making. Second, standardized report format allowed the reader to obtain more information in a short time. Detailed and transparent reporting made it easy to replicate the study and avoid the waste of medical resources.

We identified five areas where information was insufficient or inadequate in most studies. These areas are sample size, harms, allocation concealment, blinding and ITT analysis. Most importantly, the reporting quality of key methodological items was poor. Our results are in agreement with similar studies assessing the reporting quality of RCTs published in other medical journals [27-29]. All of them showed a suboptimal reporting quality, with key methodologies being usually the most poorly reported items. Allocation concealment, blinding, and ITT analysis are critical in avoiding selection, performance/detection, and attrition bias, respectively. An overestimation of treatment effects has been demonstrated in trials with inadequate key methodological design comparison with trials that adequately reported these methodological items [19].

Understanding the importance of transparency in reporting clinical trials, an international team, including epidemiologists, statisticians and journal editors, developed the Consolidated Standards for Reporting Trials (CONSORT) statement in 1996 [30]. The CONSORT statement is an evidence-based set of recommendations for reporting two-arm, parallel-group RCTs, including a minimum set of items to be reported pertaining to the rationale, design, analysis, and interpretation of the trial (that is the CONSORT checklist) and a diagram describing flow of participants through a trial (that is a flow diagram). It is intended to facilitate the complete and transparent reporting of RCTs and in turn aid in their critical appraisal and interpretation. The effectiveness of CONSORT in improving the reporting quality of RCTs has been widely evaluated. A 2008 systematic evaluation investigated whether there had been an improvement in quality of reporting for RCTs since the publication of CONSORT statements [31]. The results of this study suggest that general standards of reporting for acupuncture trials have significantly improved since the introduction of the CONSORT statement in 1996 [31]. However, the magnitude of improvement varied considerably among included studies. A possible explanation for this variability is the lack of consistency in enforcing the use of the CONSORT checklist among CONSORT adopter journals. Cobo *et al*. [32] developed a RCT to investigate the effect of an additional review based on reporting guidelines such as CONSORT on quality of manuscripts. They found that it is difficult for authors in adhering to high methodological standards at the latest research phases; to boost paper quality and impact, authors should be aware of future requirements of reporting guidelines at the very beginning of their study [32].

Interestingly, we find the overall reporting quality of RCTs in MET for PCOS is improving with the year increasing, which indicates that more and more researchers and editors are realizing the importance of reporting in RCTs due to the widely adoption and promotion of CONSORT. The effect of CONSORT in reporting RCTs of MET for PCOS still needs evaluation. A future evaluation (for example, before and after study, RCT) of the reporting quality after CONSORT endorsement would be useful in assessing the effectiveness of this measure. Meanwhile, our finding that the overall reporting quality based on the CONSORT statement was correlated with funding source, also suggested clinical trials with funding have more capacity to provide assurance for the better quality of study design and reporting of RCTs.

There are some limitations to our study. First, we didn’t directly measure RCT methodological quality, because we did not verify the information from the authors or their protocols. As important methodological criteria may be omitted in published reports although adequately carried out, the quality of reporting should be taken only as an imperfect surrogate of true methodological quality. Nevertheless published reports are the major source for clinicians to judge the validity of the results, making the quality of the report essential [20]. Second, to evaluate the quality of reporting in RCTs quantitatively, according to some rating methods published in previous studies [20-23], we extracted major items, not all items, from the CONSORT 2010 statement. Despite these limitations, we think our results have good internal validity. In our survey, the selection and abstraction processes were independently performed by two qualified assessors. Disagreements were uncommon, and they occurred often due to lack of transparency or contradictory information in the reports.

## Conclusions

Our findings show that the reporting quality of RCTs in MET for PCOS is suboptimal especially in key methodological items. Regarding the crucial methodological issues of blinding, allocation concealment, and analysis by ITT, our results stress the need for researchers involved in RCTs of MET for PCOS to improve the methodological quality of their research through a strengthened international collaboration. Reporting of RCTs on MET for PCOS should meet and keep up with the standards of the CONSORT statement.

## Abbreviations

CC: clomiphene citrate; CENTRAL: Cochrane Central Register of Controlled Trials; CONSORT: Consolidated Standards of Reporting Trials; ITT: intention-to-treat; MET: metformin; PCOS: polycystic ovary syndrome; RCTs: randomized controlled trials.

## Competing interests

The authors declare that they have no competing interests.

## Author’s contributions

BYC: conception and design, data collection and analysis, manuscript writing and final approval of the manuscript. JL: data collection and analysis, critical revision and final approval of the manuscript. CZ: revising the manuscript critically for important intellectual content and final approval of the manuscript. MYL: conception and design, manuscript writing, final approval of manuscript. All authors read and approved the final manuscript.

## Supplementary Material

Additional file 1 Database of RCTs in MET for PCOS. Click here for file
